# Notes from the Field: Epidemic Keratoconjunctivitis Outbreak Associated with Human Adenovirus Type 8 — U.S. Virgin Islands, June–November 2016

**DOI:** 10.15585/mmwr.mm6630a3

**Published:** 2017-08-04

**Authors:** Marie E. Killerby,, Matthew J. Stuckey,, Irene Guendel,, Senthilkumar Sakthivel,, Xiaoyan Lu,, Dean D. Erdman,, Eileen Schneider,, Ryan Fagan,, Michelle S. Davis,, John T. Watson,, Susan I. Gerber,, Holly M. Biggs,, Esther M. Ellis,

**Affiliations:** ^1^Division of Viral Diseases, National Center for Immunization and Respiratory Diseases, CDC; ^2^Epidemic Intelligence Service, CDC, ^3^Division of Healthcare Quality Promotion, National Center for Emerging and Zoonotic Infectious Diseases, CDC; and ^4^U.S. Virgin Islands, Department Of Health.

On October 11, 2016, the U.S. Virgin Islands Department of Health (USVI DOH) was notified by a local ophthalmologist of an unexpected increase in the number of patients with suspected epidemic keratoconjunctivitis (EKC) during the preceding month. EKC is a severe form of acute conjunctivitis caused by human adenoviruses (HAdVs). Clinical illness typically lasts 1 to 3 weeks and is usually self-limited; treatment is supportive (1). HAdVs can survive for weeks in the environment and are resistant to common disinfectants (*2**,**3*). USVI DOH and CDC investigated during October 11–November 29, 2016 to determine the scope of the outbreak, and provide infection control recommendations.

A case of EKC was defined as 1) a diagnosis by an ophthalmologist or optometrist of EKC, adenoviral conjunctivitis, or viral conjunctivitis (excluding conjunctivitis diagnosed in association with presumed Zika virus infection); or 2) laboratory confirmation of HAdV type 8 (HAdV-8) from a specimen collected by conjunctival swab in a person on the affected island during June 1–November 29, 2016. A health care–associated case, was defined as a case in a person who had visited an eye care practice ≤14 days before onset of symptoms.

Available medical records were reviewed for patients with diagnoses of acute conjunctivitis during June 1–November 4, 2016 from all six eye care practices on the affected island. Additional cases were identified prospectively through collection of conjunctival swabs from patients evaluated for acute conjunctivitis at eye care practices, a hospital emergency department, and two family practice clinics during October 14–November 29, 2016.

Environmental testing was conducted, and routine infection control practices were assessed at the two eye care practices where health care–associated transmission was suspected to have occurred: the initial reporting practice (practice A) and a second eye care practice (practice B). Environmental samples were collected from eye care equipment and high-touch surfaces in waiting areas and patient examination rooms. Conjunctival and environmental swabs were tested at CDC using HAdV real-time polymerase chain reaction (qPCR). Positive specimens were molecularly typed, based on sequencing of hexon hypervariable regions 1–6 and inoculated into A549 cells for virus isolation. Whole genome sequencing was performed on five selected cell culture isolates, obtained from patients who were infected at the beginning, middle, and end of the outbreak.

Seventy-eight cases were identified in patients from four eye care practices, two family practices, and the hospital emergency department. The median patient age was 45 years (range = 9 months–90 years), and 33 (42%) were men. Ocular signs and symptoms included redness (68%), watery discharge (50%), and pain (29%). Severe signs included corneal infiltrates (17%) and pseudomembranes (6%). At least 12 cases (15%) were health care–associated (Figure). One health care–associated case occurred in a health care worker. Seventeen patients whose infections were not health care–associated reported a symptomatic household or community contact. Among 45 conjunctival swabs available for testing, 19 (42%) were positive for HAdV-8. Genome sequences obtained from five HAdV-8 isolates were 100% identical with one another and showed 97.7% (accession number AB861610.1) to 99.9% (accession number KT340070.1) nucleotide sequence similarity to other HAdV-8 genome sequences available in GenBank, the National Institutes of Health genetic sequence database. Among 39 environmental samples collected from practice A, eight (21%) were positive for HAdV from the following surfaces or devices: a doorknob, an eye occluder, phoropter (refractor), an examination light, two hand sanitizer dispensers, a bathroom faucet, and the surface of a multiuse eye drop bottle. Among 10 environmental samples collected from practice B, two (from a phoropter and a waiting room chair) were positive for HAdV. Environmental samples were unable to be typed because of the low viral load present. Nine HAdV positive environmental swabs were inoculated into A549 cells, and no cytopathic effect was observed after one blind passage, therefore the presence of live virus could not be confirmed. Observed gaps in infection control at the practices A and B included use of disinfectants without a proven efficacy against HAdV.

**FIGURE:**
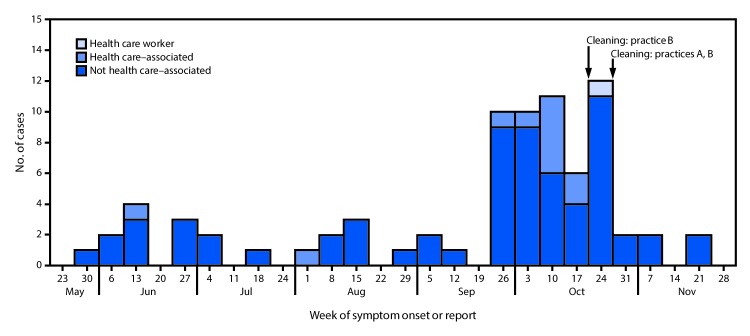
Cases of epidemic keratoconjunctivitis* (N = 78), by date of symptom onset or report — U.S. Virgin Islands, June 1–November 29, 2016 * Diagnosis by an ophthalmologist or optometrist of epidemic keratoconjunctivitis, adenoviral conjunctivitis, or viral conjunctivitis (excluding conjunctivitis diagnosed in association with presumed Zika virus infection) or laboratory confirmation of human adenovirus type 8 from a specimen collected by conjunctival swab during June 1–November 29, 2016. A health care–associated case was defined as a case in a person who had visited an eye care practice ≤14 days preceding symptom onset.

Infection prevention and control guidance was provided to six eye care practices and the emergency department. Recommendations included 1) maintaining proper hand hygiene, 2) cohorting patients with suspected EKC in the health care setting, 3) refraining from using contents from eye drop bottles for more than one patient, 4) using an Environmental Protection Agency–registered disinfectant with proven activity against HAdVs to decontaminate surfaces and equipment, and 5) furloughing symptomatic employees (*1**,**2*). Thorough cleaning according to recommendations was performed at both practice A and practice B, after which reports of EKC declined markedly (Figure). Patient education resources were provided to six eye care practices and the hospital emergency department to support prevention of community spread. No further reports of EKC were received after December 31, 2016.

Health care–associated transmission of EKC in this outbreak highlights the importance of infection control in eye care practices, including the use of disinfectants with proven efficacy against adenoviruses. The occurrence of household transmission also underscores the importance of patient education regarding measures to prevent the spread of EKC.
